# A Multisensory Multilevel Health Education Model for Diverse Communities

**DOI:** 10.3390/ijerph16050872

**Published:** 2019-03-10

**Authors:** Olajide Williams, Ewelina M. Swierad

**Affiliations:** Department of Neurology, Columbia University Medical Center, New York, NY 10032, USA

**Keywords:** Socio-Ecological Model, public health education, Stroke Education, health communication

## Abstract

Owing to their enormous capacity to improve health and save lives, public health researchers and practitioners have worked on developing effective frameworks for the optimization of health promotion strategies. A multilevel focus, as exemplified by the Socio-Ecological Model (SEM), is one common denominator among these frameworks. The SEM highlights important social and ecological influences on health behavior by delineating the different levels of influence. These include public policy, organizational, community, interpersonal, and intrapersonal levels, which, when considered during the development of health promotion campaigns—especially those that focus on health education—strengthen the influence of that campaign on targeted behaviors. However, the SEM lacks a complimenting framework for understanding the role of conventional and unconventional approaches to health education; that is, how to design a health education intervention that considers both the context, such as the social and ecological levels of influence, and the best approaches for developing and delivering the health education in a manner that optimizes its effectiveness in today’s modern and increasingly diverse world. Addressing this gap, the current article presents an integrative Multisensory Multilevel Health Education Model (MMHEM), which incorporates three key domains—(1) Art (innovativeness/creativity), (2) Culture (cultural tailoring), and (3) Science (evidence-based), while promoting the importance of considering the socio-ecological levels of influence on targeted behaviors. Using a successful health education intervention, called the Hip Hop Stroke, we deconstruct the Multisensory Multilevel Health Education Model and discuss its potential role as a guide for developing public health education interventions.

## 1. Introduction

Mechanisms behind effective public health education remain complex, and the best practices are seldom implemented. Consequently, too many health education programs, especially those that target high poverty, low literacy communities are unsustainable or unimpactful [1]. Moreover, the excessive quantity of information from traditional and social media, coupled with the overwhelming volume of commercial advertising, has led to information overload and intense competition for the public’s attention [2]. These factors are responsible for what has been called “the attention economy”, in which human attention is characterized as a commodity that is becoming increasingly scarce [3]. Therefore, novel approaches to health education in this modern environment has become a public health imperative, especially among economically disadvantaged populations where existing disparities are the highest [4].

A body of well-designed programs has been shown to improve health outcomes [5,6,7,8]; however, the penetration of these programs into an economically disadvantaged population is often inadequate, and their sustainability is a major challenge. To date, an integrative approach to health education that considers both conventional and unconventional (those that incorporate new media, entertainment, and other art forms) methods has not been systematically explored, even though such an approach may hold promise, particularly for health education programs within diverse communities. 

Current frameworks focus on constructs governing “why” a person engages in a certain behavior and consequently “what” needs to be a target of an intervention designed to address this behavior. They include (1) individual factors, such as attitudes, motivation, normative beliefs, and self-efficacy; (2) interpersonal factors, such as social norms and social support; and (3) constructs that target the different stages of behavioral change [9]. For example, Health Belief Model [10] and Theory of Planned Behavior [11] address individual factors that affect one’s health, such as attitudes, motivation, or perceived behavioral control, just to name a few. Social Cognitive Theory [12], Elaboration Likelihood Model [13], or Social Support Theory [14] build on individual models and consider social factors that influence one’s health including social support, modeling, and persuasion. The most recognized behavioral stage models—Transtheoretical Model [15] and the Diffusion of Health Promotion Innovation Model [16]—are predominantly concerned with stage-wise, step-by-step progression of adopting a healthy behavior. Finally, the Educational Entertainment concept [17] focuses on utilizing different forms of art and entertainment as educational and communication tools. Although these frameworks represent valid and well-established approaches to health education, they tend to focus on constructs governing the “why/what” of human behavior, and lack specific strategies to help guide the design and implementation of effective health campaigns, which we refer to as the “how” of health education.

In addition to frameworks that address the “why/what” of human behavior, public health practitioners have recognized the importance of context in human health. Indeed, most successful programs consider the major social and environmental factors influencing behavior. We refer to these socio-ecological influencers as the “who/where” of health education. These levels of influence, which are expressed in the Socio-Ecological Model (SEM), [18] include: (1) public policy, (2) organizations/institutions within the community, (3) community culture and social norms, (4) family and friends, and (5) factors that are intrinsic to the individual [19,20]. However, while the Socio-Ecological Model (SEM) focuses on which level of the human environment (i.e., physical and social or “who/where”) needs targeting, it does not address the crucial question of “how” public health practitioners can best design effective health education strategies that permeate these levels. We attempt to answer the “how” question by presenting a functional framework outlining the dynamic relationship between the health education strategies and their levels of influence within individuals’ socio-ecological landscape. This framework—the Multisensory Multilevel Health Education Model (MMHEM)—represents an integrative health education model that provides practitioners with key constructs along with their operationalizable units for public health practice. Our model incorporates elements from both conventional and unconventional health education approaches. Indeed, by integrating unconventional strategies, such as leveraging culturally tailored artistic materials in the form of storytelling, music, animation, film, gamification, among others, our model moves beyond traditional approaches to health education.

The current article presents a Multisensory Multilevel Health Education Model (MMHEM) that encompasses the (1) “why/what”, (2) “who/where”, and (3) “how” of health education. The “why/what”, which reflects mechanisms governing human behavior, is often the target of existing health behavior models (e.g., attitudes, motivation, normative beliefs, self-efficacy, social norms, social support, etc.); the “who/where” reflects the levels of influence as outlined by the Socio-Ecological Model (e.g., family, community, organizations, public policies); and, the “how” considers methodological and innovative approaches to health education design, cultural adaptation, and implementation.

We deconstruct the MMHEM through the prism of an evidence-based intervention called Hip Hop Stroke (HHS)—a multisensory, culturally tailored, evidence-based intervention designed to increase stroke recognition and 911 calls (stroke preparedness) among urban minority children and their families [21,22,23,24,25]. Due to the disproportionately high stroke case fatality rates among United States (U.S.) Blacks, and the correspondingly low acute stroke treatment rates, HHS was developed to increase the stroke treatment rates among this group. Acute stroke treatment is a time-dependent activity, because these life-saving treatments can only be administered to patients within 4.5 h from the onset of stroke symptoms. Unfortunately, among U.S. Blacks, stroke patients are often excluded from these treatments due to late hospital arrival, which, in-turn, has been linked to behavioral failure to act in an urgent manner when the symptoms begin (lack of “stroke preparedness”). HHS is designed for children (direct targets) because of the high proportion of children living with older high-risk parents/grandparents (indirect targets) in economically disadvantaged Black communities [26]. These children, who may be the only witnesses present during a stroke event, are trained to act as first responders by immediately calling 911 upon witnessing a stroke in their home. They are also trained to serve as a vehicle through which stroke symptoms knowledge and their urgency are delivered to parents and grandparents at home. Hip Hop Stroke uses a novel multisensory, multimedia, and culturally tailored strategy to increase the appropriate behavioral action, which is to immediately call 911 in the event of a stroke, and thereby improve time-dependent acute stroke treatment rates. Importantly, HHS has been shown to be effective in a large randomized clinical trial (RCT) [25].

## 2. The Multisensory Multilevel Health Education Model

### 2.1. Socio-Ecological Model (SEM) and Health Education: Foundation of the Multisensory Multilevel Health Education Model (MMHEM)

Just as the English poet John Donne emphasized that “no man is an island entire of itself; every man is a piece of the continent, a part of the main”, the Socio-Ecological Model (SEM) affirms that individuals exist within a broader environmental and social ecosystem that shapes their health behaviors [18]. Recent data suggest that the more levels of influence targeted by a health campaign, the greater the probability that individuals will improve their health behaviors [27]. Lewis and colleagues [28] examined whether the implementation of multilevel interventions, in the case of diabetes self-management, optimizes the effects of the interventions. They found that combining interventions on different levels of the SEM enhanced diabetes self-care among patients [28]. This effect was attributed to a “convergence strategy”*—*the idea that multilevel interventions reinforce each other by changing the patterns of interactions between the stakeholders that are involved in a particular health promotion endeavor. Although the benefits of multilevel interventions are clear, health education interventions often target a single level of influence; this singular focus results in missed opportunities to leverage the “convergence strategy” [29]. Therefore, we propose a Multisensory Multilevel Health Education model that facilitates the a priori consideration of the different (applicable) levels of behavioral influence in the design of health education interventions, and we discuss its major domains.

### 2.2. Art, Culture, and Science—Major Domains of the Multisensory Multilevel Health Education Model

**Figure 1:**
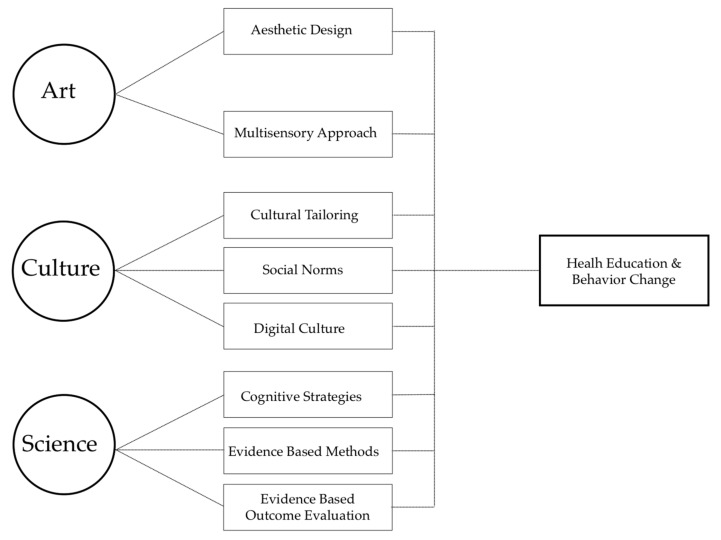
The Multisensory Multilevel Health Education Model.

One way to design health education programming is through the use of art (innovativeness/creativity), culture (cultural tailoring), and science (evidence-based) (see Figure 1). From the art perspective, researchers have long recognized the parallel between health communication and the fields of creative marketing such as various forms of advertisements [30,31,32]. Despite their differences, the goal of health education and marketing is similar—that is to influence behavior (health behavior or consumer behavior) in an innovative, effective, and sustainable manner [30]. In addition to the art domain, health programs need to consider an individual’s culture [33]. This consideration is particularly important in the case of campaigns targeting people from culturally diverse communities [34] where social norms interact with the behavior change being sought. In fact, culturally discordant programs contribute to the failures of many health campaigns [35]. Finally, health education programs need to be guided by scientific evidence supporting their content, followed by a rigorous evaluation of their effectiveness prior to dissemination [36].

**Figure 2:**
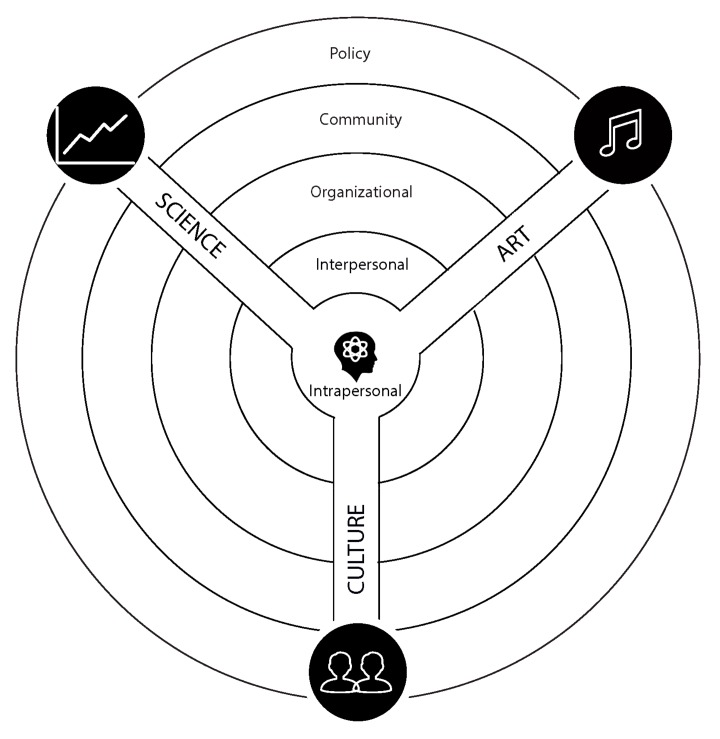
The Multisensory Multilevel Health Education Model (MMHEM) integrates art, culture, and science into multiple levels of influence of the SEM (intrapersonal, interpersonal, organizational, community, and policy).

The MMHEM is a conceptual framework designed to provide guidance for the development of health education interventions. It comprises three major domains—art, culture, and science—and eight subdomains (see Figure 1 & Table 1). The model represents an integrative approach to public health education operating at different levels of behavioral influence (see Table 1). Its application should be considered when developing and implementing public health interventions, especially those that seek to permeate multiple levels of the SEM (see Figure 2).

#### 2.2.1. Art

The art domain of the MMHEM pertains to health education interventions that are multisensory and aesthetically sensitive. They leverage visual (iconic), auditory (echoic), tactile, and kinesthetic sensory inputs in their design through the creative arts (see Table 1). This multisensory focus may help health education programs compete more effectively with other industries in the “attention economy” environment. The addition of an interactive feature to this domain serves as a synergistic attribute for stimulating multiple senses [37]. Examples of the art domain includes the use of music, storytelling, pictographs, multimedia, and movement (see Table 1). Although health education programs may focus on one or two of the above examples in their design, research on multisensory learning suggest that education strategies combining multiple senses to convey a single message are more effective than strategies using dual or single sensory modalities [38,39,40,41]. For example, individuals remember more if they are presented with a drawing of a common object that is paired with a semantically congruent sound (e.g., picture of a bell and a sound “dong”), as opposed to a drawing alone [39]. Similarly, from a consumer behavior perspective, multisensory advertisements of food are more effective than advertisements focused on taste alone [42].

Since perception and memory are multisensory in nature, MMHEM promotes the integration of visual, auditory, and kinesthetic strategies into health messages. From a neuroscience perspective, integrating congruent multisensory information enhances individuals’ performance, learning, decision-making [43], and memory [44]. Indeed, human memory—encoding, storing, and retrieving information—is accustomed to function in a multisensory environment [44]. For example, learning a new language through multiple senses (i.e., visual, auditory, kinesthetic) outperforms verbal learning alone [45]. In fact, such cross-sensory interactions occur at very early stages of human perception [46]. Moreover, the human brain is organized to process information coming from different sensory channels concurrently in order to form a comprehensive picture of reality [47]. In a series of experiments, Seitz, Kikm, and Shams [48] found that integrating sound and visual learning yielded more effective acquisition of new skills than single-sensory modality learning, provided that the audio and visual messages consisted of congruent information. Similarly, combining text with semantically congruent visual images improved learning [49]. These data support the notion of a multisensory “congruency effect”, suggesting that semantically congruent multisensory stimuli improve the encoding of new information and enhance its later recognition memory [50].

In the context of health education, evidence suggests that cartoon illustrations and pictographs are more successful than written materials alone for improving patient recall and comprehension of consent materials, particularly among low literacy populations [51,52]. In the same vein, George and colleagues [53] found that animated videos might be effective educational tools for increasing the health literacy of minority populations. However, despite this recent surge in interest regarding the application of multisensory and multimedia art forms to public health, the best way of designing these programs in a manner that optimizes effectiveness remains unclear [54].

#### 2.2.2. Culture

The culture of a community or primary audience needs to be considered across the entire spectrum of health education design and implementation [33,55] (see Table 1). Cultural factors include cultural tailoring, alignment with social norms, and digital culture (e.g., social media proclivity of the community). Health education programs can strive to create connections between the healthy behavior of interest and important social and cultural identities (Identity Based Motivation theory) [56]. Such cultural tailoring promotes the relevance of the intervention to the local community through programing that resonates with culturally accepted norms, beliefs, and values [57]. Similarly, according to Identity Based Motivation theory, people prefer to act in ways that conform to their most cherished identities, such as race, ethnicity, gender, and social class that can be primed through identity-congruent situational cues (e.g., culturally tailored health messages) [58]. Therefore, designing health interventions that create connections between the target behavior and important social and cultural identities [56] is necessary for their acceptance, success, and sustainability.

The role of digital culture, particularly social media in health promotion, is a rapidly evolving and growing area of interest for public health practitioners [59], mainly because of social media’s widespread use, reach, and cost-effectiveness [60,61]. Although more evidence is needed to determine the effectiveness of this approach to improving health among technology-averse groups, such as the elderly [62], recent reviews suggest that social media may not only be effective for promoting health among the youth, but also among economically disadvantaged populations, which may support its use as an adjunctive tool for promoting health equity [63]. Moreover, in the case of young, technology savvy audiences, it is critical to consider digital culture, particularly social media usage, in the design of health education interventions [57]. As demonstrated by recent research, the popularity of different social media platforms depends on individuals’ age and gender [61]. While Facebook and YouTube remain relatively popular across all age groups, Snapchat and Instagram are favored by younger generations, and Pinterest is significantly more popular among women than among men [61]. It is therefore unsurprising that social media platforms have become attractive venues for health education, and the MMHEM may serve as a framework for their systematic inclusion.

The concept of “identity signaling” [64] can also be used in the design and evaluation of the cultural appropriateness of public health interventions, especially those targeting the youth. Identity signaling is based on social and biological markers individuals can identify with, and the meaning associated with these identity markers [64]. Consequently, if people identify or want to identify with a given identity, they may adapt their behaviors to conform to these identities [65,66]. For example, if teenagers believe that “cool kids” drink and smoke, they may emulate these negative behaviors in order to be perceived as “cool”. Conversely, if the same teenagers believe that “cool kids” engage in healthy habits, they may emulate the positive behaviors. These approaches help create and consolidate new norms and identities, which may spread through social networks and establish target behaviors as cool [67,68]. As a result, new healthy social norms are created, promoting health behaviors that become habitual and normative within a given cultural environment [67,68].

Identity signaling and shaping healthy social norms can also be achieved through music. Given that music assumes as many forms as culture, it is a venue through which health interventions can express a particular cultural identity and social norms [69]. In the context of health education, it is critical to select a music genre that resonates with a given cultural group [69]. For example, the style of music and lyrics may convey a particular cultural meaning, generating a comfortable feeling of familiarity [69,70]. In fact, listening to culturally familiar music, in contrast to culturally unfamiliar music, generates greater levels of brain activation similar to patterns observed when listening to a familiar language [71]. Series of studies have demonstrated that culturally familiar music is more memorable than culturally unfamiliar music [72]. This phenomenon may be related to the fact that music activates related knowledge structures and primes a person’s understanding of the world, including their cultural environment [73,74].

#### 2.2.3. Science

The science domain of the Multisensory Multilevel Health Education Model highlights the importance of utilizing evidence-based methods and evidence-based outcome evaluations (see Table 1). The model also promotes the incorporation of strategies for optimizing cognitive processes linked to memory and learning. Moreover, every decision involved in the design, implementation, and evaluation of a public health education intervention needs to be made based on the best available scientific evidence [75] and then rigorously evaluated prior to dissemination.

Example of design flaws in public health education can be found in its most basic component: the message itself. Although decades of marketing research have revealed key factors that make messages memorable and shareable, these attributes are infrequently considered upfront. Chip and Dan Heath [76] found several properties in message design that improves the “stickiness” (memorability) of a message. These properties help messages to attract attention, enhance memorability, and facilitate learning, and they include: (1) simplicity, (2) unexpectedness, (3) concreteness, (4) credibility, and (5) emotional narratives [76]. Regarding the shareability or “contagiousness” of a health message [77,78], studies suggest that the message needs to be either interesting or useful [78]. Factors that increase the contagiousness of a message include: social currency, triggers, emotional content, and practical value [78] (see Table 1).

The MMHEM emphasizes the critical role of Evidence Based Methods and Outcome Evaluation in the design, implementation, and evaluation of health education programs. This is because, unlike other forms of medical interventions, such as testing a new drug, device, or procedure, public health interventions are not usually held to the same evidence-based standards prior to dissemination and implementation. The MMHEM encourages health educators and researchers to design health interventions that are based on the most robust up-to-date evidence, methods, measures, and knowledge. What constitutes an evidence-based intervention is well established in the literature [79]. This literature focuses on three types of evidence: type 1 evidence is research that identifies the magnitude, severity, and preventability of a health problem. Type 2 evidence recognizes the most effective, evidence-based health or behavioral interventions addressing the problem [79]. Based on the methodological quality and validity of the research supporting the intervention, several additional criteria have been established. An example of one such criteria is the “level of effectiveness rating scheme”, which places adequately powered randomized control trials at the highest level (level 1) due to their control of confounding factors and bias, unlike case studies/case series with no control groups and expert consensus opinions, which have the lowest level of evidence (level 5) supporting their effectiveness [80]. Although it may not be feasible to test all of the interventions in a randomized control trial, it is important to apply the highest level of evidence to the design and implementation of health education programs to ensure that they are as unbiased and unconfounded as possible. Finally, type 3 evidence focuses on how the problem should be addressed, including specific information regarding how to design and implement health interventions, the social and physical context of the intervention, and the methods and measures that are used for outcome evaluation [81]. 

As presented in the MMHEM (see Table 1), in order to enhance the quality of evidence-based methodology, (a) scientific advisory boards can help provide essential expertise regarding the particular health problem being targeted by the health education program; (b) literature reviews/subject matter experts can help enhance the understanding of specific elements of the problem; (c) best practices derived from successful programs should be implemented; (d) researchers and practitioners should employ conceptual frameworks to guide the design, implementation, and evaluation of their interventions; and, (e) multidisciplinary teams can be formed to provide a broad scope of knowledge and skills necessary for different components (such as the art, culture, and science components) of effective health education. Regarding outcome evaluation, evidence-based methods should include both psychometrically reliable and validated outcome measures and rigorous research designs to test the efficacy and effectiveness of the intervention.

### 2.3. The Interaction between Art, Culture, and Science

All of the domains incorporated in the MMHEM dynamically interact with each other. In other words, one domain alone, without considering the other two domains may not be sufficient for increasing the effectiveness of a particular health intervention. For example, when designing health education programs for reducing childhood obesity among African American children, health practitioners need to simultaneously consider effective multisensory strategies (art) that are culturally relevant to the particular cultural group (culture) and are based on the best available research evidence (science). Therefore, all three domains—art, science, and culture—co-exist in a dynamic and symbiotic relationship, and their mutual influence needs to be considered at each stage of health education design, implementation, and evaluation.

## 3. Hip Hop Stroke: An Illustration of MMHEM

### 3.1. General Overview of HHS

Hip Hop Stroke (HHS) was developed to improve stroke recognition and calling 911 among economically disadvantaged ethnic minority children and their parents [23]. It is an example of a multisensory and multilevel public health education intervention. HHS leverages the science behind multisensory learning through its musical, multimedia and interactive modules [23], providing opportunity for physically dynamic programming that involves visual, auditory, and also kinesthetic senses. By incorporating art, culture, and science, HHS teaches children the signs and symptoms of stroke, the urgency of calling 911, and the time-dependent nature of acute stroke treatment. These “stroke prepared” children are then motivated to share stroke information with their parents through an approach called “Child Mediated Health Communication” [24], which is based on homework activities. In a randomized HHS clinical trial that included more than 3000 children and 1000 parents, the main outcome measure was stroke preparedness as measured by (1) a valid instrument used to assess stroke preparedness of children and parents, and (2) a questionnaire administered to parents to assess knowledge of the F.A.S.T. acronym (i.e., Facial drop, Arm weakness, Speech slurring/disturbance, and Time to call 911) [25]. HHS was shown to improve the stroke preparedness of children and parents. Although there was no mediation effect between the children and their parent’s stroke preparedness scores, parents of children who shared stroke information with them showed a greater increase in stroke preparedness over time than parents whose children did not share stroke information, supporting the “child mediated health communication model” [25]. In addition, several intervention children called 911 for real-life stroke occurrences among family members. These real-world stroke cases demonstrate that the HHS intervention not only improves stroke preparedness of children, it also translates into real behavior change. HHS is now being disseminated across New York State (NYS) in a policy-relevant partnership with the NYS department of health and 47 hospitals have adopted it.

### 3.2. Art Domain of Hip Hop Stroke

**Figure 3:**
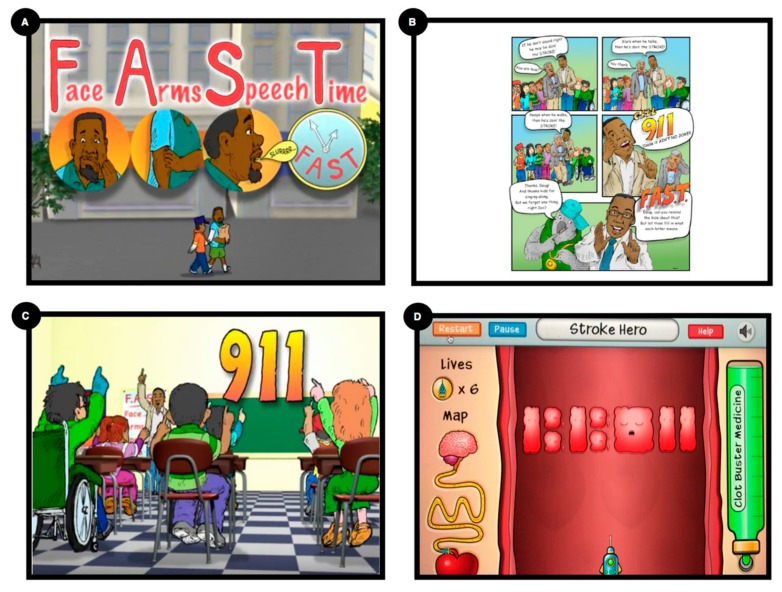
Sample Hip Hop Stroke Media. (**A**) “Keep Your Brain Healthy”—animated feature showing the Facial drop, Arm weakness, Speech slurring/disturbance, and Time to call 911 (F.A.S.T.) mnemonic; (**B**) Hip Hop Stroke Comic Book; (**C**) “Stroke Ain’t No Joke”—animated feature; and (**D**) “Clotbuster” video game.

Developed using Entertainment Education (EE) concepts [17], the HHS modules are comprised of the following multisensory tools: hip hop music (auditory and kinesthetic), animated narrative cartoons (visual/storytelling), a video game (interactive), and a comic book (visual/storytelling) (see Figure 3). One of the cartoons, *Stroke Ain’t No Joke* (https://hhph.org/resources/stroke-aint-no-joke-2/) is a musical narrative, “role play” cartoon that teaches stroke recognition and action through the use of the F.A.S.T. acronym and the therapeutic benefit of early treatment (i.e., “time is brain”) (see Figure 3) [22]. The importance of the “time is brain” message is encapsulated in the “Stroke Hero” “clotbuster” video game, which allows children to navigate a clot-busting spaceship within a human artery in order to shoot down blood clots that occlude the passage of blood to the brain (stroke pathophysiology and treatment). A second cartoon, *Keep Your Brain Healthy*, is a musical narrative “role play” cartoon that teaches children the relationship between stroke risk factors (e.g., diabetes, hypertension, smoking, poor diet, sedentary life) and stroke occurrence, and empowers them to positively influence their parents’ behavior.

### 3.3. Culture Domain of Hip Hop Stroke

The development of HHS incorporated elements from cultural adaptation frameworks, such as the ecological validity model [82] and utilized community engagement techniques (e.g., focus groups, semi-structured surveys, iterative development, pilot studies) [23]. Educational materials were designed in collaboration with school children and then pilot-tested for acceptability and efficacy. The HHS intervention also incorporated the concept of identity signaling. This was accomplished by incorporating into narrative plots, cartoon characters representing different sexes (i.e., male, female), as well as race and ethnicity (i.e., black, brown, and white kids), and even disability status (i.e., a kid in a wheelchair). As a result, children could identify with them and feel represented. HHS then promoted the image of “cool kids” engaging in healthy behaviors. In addition, hip hop music in HHS interventions was strategically selected because it represents the most popular music genre among the ethnic groups for whom HHS was designed (Nielson Media).

Finally, although not used in HHS, a Narrative Performance Scale [83] can be utilized to culturally adapt a narrative based media tool by evaluating three components of the health message: interest, realism, and identification [83]. Interest is the degree to which recipients pay attention to the health message; realism is the extent to which the audience perceives the plot as authentic; and identification is the degree to which health message recipients identify with the characters portrayed in the health message [83,84].

### 3.4. Science Domain of Hip Hop Stroke

Scientific evidence supporting HHS was derived from the education, marketing, psychology, neuroscience, and stroke literature, as well as from pilot projects. These include: (1) theoretical underpinnings of HHS that utilized Social Cognitive Theory and Theory of Planned Behavior/Reasoned Action [11,12]; (2) methods used to enhance memorability (“stickiness”) [76] and shareability (“contagiousness”) [77,78] of HHS messages; (3) the minimum frequency of exposure (effective frequency) to HHS core media [85]; (4) methods used during measurement and data collection [86,87]; (5) the time-dependent nature of stroke treatment [88]; and, (6) the use of a randomized controlled trial design to test effectiveness prior to dissemination.

HHS messages were designed to be “sticky” through the use of rhythm, rhyme, repetition, and a simple four-letter actionable acronym that is embedded in different media modules (see Figure 3). These messages are also “unexpected” (counterintuitive messages are often more memorable), such as the joint appearance of an animated hip hop artist and an animated medical doctor rapping about stroke symptoms. Moreover, the use of a well-known hip hop artist and a real medical doctor enhances the “credibility” of the message, while the inclusion of real life “emotional stories” increases the message memorability.

Regarding contagiousness or shareability, HHS children receive a fridge magnet that contains the F.A.S.T. acronym message. This magnet serves as a constant reminder or “trigger” of stroke recognition and the need to call 911, if a stroke occurs. The usefulness or the “practical value” of this life-saving knowledge is highlighted through concrete animated role-play depictions of children using this knowledge to save the life of a stroke victim. These “emotional stories” delivered across diverse HHS media enhances the contagiousness of the message, while the program’s Child-Mediated Health Communication (CMHC) Framework [24] facilitates real world sharing of the message with parents and grandparents at home.

### 3.5. Levels of Behavioral Influence and Hip Hop Stroke

**Figure 4:**
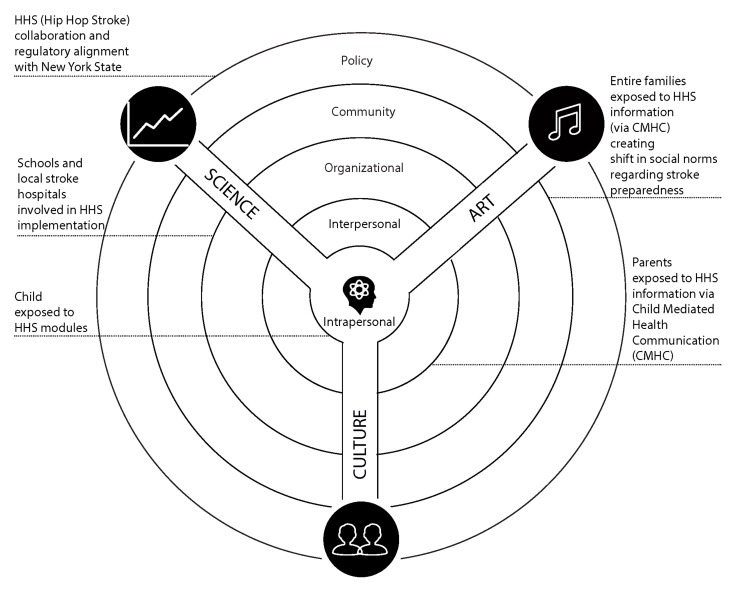
Hip Hop Stroke and the Socio-Ecological Model illustrated through the Multisensory Multilevel Health Education Model. Legend: CMHC = Child Mediated Health Communication; HHS = Hip Hop Stroke.

Hip Hop Stroke combines art, culture, and science across multiple levels of the Socio-Ecological Model. At the intrapersonal level, children are exposed to three one-hour modules through an online HHS portal in school classrooms. Through Child-Mediated Health Communication [24], the children share stroke information with their parents (interpersonal level). Thus, entire families become “stroke prepared” and this “preparedness” begins to shift social norms regarding stroke action behaviors and their urgency (community level). At the organizational level, HHS engages schools and stroke hospitals that serve as intervention sites (the former) and dissemination hubs (the latter). Finally, at the policy level, HHS leverages the regulatory need for stroke hospitals to engage in local community stroke education through a formal collaboration with the New York State Department of Health Stroke Designation Program. An illustration of the Hip Hop Stroke interventions across all levels of Socio-Ecological Model is provided in Figure 4.

## 4. Discussion

HHS is an example of a public health education intervention that deploys art, culture, and science across multiple levels of an individuals’ physical and social environment. Our experience with HHS and evidence from its randomized trial [25] suggests that the effectiveness of public health education campaigns can be optimized through innovative, culturally tailored, and evidence-based multisensory and multilevel approaches.

The MMHEM is built on well-established health education frameworks, predominantly the Socio-Ecological Model and Entertainment Education theory. However, several unique features distinguish MMHEM from the existing health education models: (1) it represents an integrative approach to health communication by combining conventional and unconventional methods of health education; (2) it outlines specific components of health communication and their targeted functions; (3) it provides an operational roadmap for the design, implementation, and evaluation of effective and multisensory health education programs; (4) it may help enhance the competitiveness of health education interventions in the “attention economy” of the modern world by mimicking persuasive and creative marketing approaches through its art domain; and, (5) it is a translational framework that enables researchers and practitioners to apply behavioral theory into practice by outlining the key steps involved.

In summary, the MMHEM integrates: (1) “why/what”, (2) “who/where”, and (3) “how” of an effective health education. It does this by highlighting specific and modifiable reasons for individual-level knowledge gaps, interpersonal barriers, and facilitators of health education (“why/what”); the socio-ecological levels of influence (“who/where”); and, the methodology of effective health education programming (“how”). When compared to existing health education models (see Table 2), the MMHEM not only incorporates key constructs of several traditional frameworks, it goes a step further by deconstructing how its major domains—art, science, and culture—can be operationalized in a manner that synergizes with people’s social and physical environments and engages their individual health behaviors. We note that the concept of educational entertainment [17] overlaps with the MMHEM in terms of its emphasis on different forms of art and entertainment as educational and communication tools. However, MMHEM expands on the educational entertainment framework by grounding these constructs within the socio-ecological context, culture, and science.

Although the Multisensory Multilevel Health Education Model is a promising framework to help guide the design and implementation of health education programs, several limitations of the model exist. First, the key constructs of the model have not been prospectively tested; rather, the MMHEM is the product of a retrospective analysis of a single, successful, widely adopted, evidence-based health education intervention, and literature review. Second, since the MMHEM is limited to a single stroke education campaign, it is important for public health practitioners to consider its applicability to their domains of interest, and the specific interventions designed to address them. Indeed, targeting multiple levels of socio-ecological influence is not always indicated or appropriate, and certain interventions may only require single level targeting. Finally, because we have not prospectively measured the specific constructs of the MMHEM, our deconstruction of HHS represents a qualitative illustration that requires validation. This may include the development of specific scales for measuring the key constructs of the MMHEM.

## 5. Conclusions

This article describes the important role of multisensory and multilevel approaches to public health education interventions and the need to frame these interventions within the social and ecological levels of behavioral influence. We posit that health education interventions targeting diverse communities need to incorporate three domains—art, culture, and science—to guide their design, implementation, and evaluation, and to improve their competitiveness in a modern world where information overload has become pervasive, and human attention is an increasingly scarce commodity.

## Figures and Tables

**Table 1:** The Multisensory Multilevel Health Education Model—Domains, Subdomains, Examples, Functions, Socio-Ecological Model (SEM) Level of Influence.

Domains	Subdomains	Examples	Functions	SEM Level of Influence
Art	1. Aesthetic Design 2. Multisensory Approach (visual, auditory, tactile kinesthetic)	(a) Music (b) Storytelling (c) Multimedia (e.g., animation, video) (d) Pictographs (e) Gamification (f) Acting (dramatization) (g) Movement (e.g., dance) (h) Virtual Reality	Enhance attention Facilitate immersion Trigger emotions Inspire action Overcome illiteracy barriers Enhance learning	Intrapersonal Interpersonal
Culture	1. Cultural Tailoring 2. Social Norms 3. Digital Culture	(a) Qualitative research (e.g., focus groups, community advisory boards, use of a narrative performance scale) (b) Cultural adaptation frameworks (e.g., ecological validity model) (c) Identity Signaling (d) Role of social media and digital platforms	Increase cultural relevance Increase personal relevance Facilitate acceptance Leverage social networks through digital platforms	Intrapersonal Interpersonal Organizational Community
Science	1. Cognitive Strategies (memory and learning)	(a) Stickiness (including the properties of a sticky message) (b) Contagiousness (including the properties of a contagious message) (c) Repetition (d) Rehearsal (e) Role Play (f) Rhymes (g) Acronyms	Facilitate learning Facilitate retention	Intrapersonal Interpersonal Organizational Community
	2. Evidence Based Methods	(a) Scientific Advisory Boards (b) Literature reviews/Subject Matter Experts (c) Best practices (d) Use of Conceptual Frameworks (e) Multidisciplinary or Transdisciplinary teams	Ensure scientific rigor including strong scientific premise	Intrapersonal Interpersonal Organizational Community
	3. Evidence Based Outcome Evaluation	(a) Psychometrically Reliable and Validated Outcomes Measures (b) Rigorous Research designs to test for Efficacy and Effectiveness	Facilitates internal and external validity	Intrapersonal Interpersonal Organizational Community Policy

**Table 2:** The comparison of MMHEM to the existing evidence-based health education models and their major focus.

Health Education Framework	*WHY/WHAT* (e.g., Self-efficacy, Perception, Normative beliefs, Persuasion, Social Norms etc.)	*WHO/WHERE* (i.e., Individuals, Family/Friends, Community, Organizations, Public Policy)	*HOW* (i.e., the Incorporation of Art, Cultural Adaptation, Scientific Evidence based Methods)
Existing Models of Health Education * (individual, interpersonal, stage-based frameworks)	×		
Socio-Ecological Model		×	
Educational Entertainment	×		×
MMHEM	×	×	×

* Health Belief Model; Theory of Planned Behavior; Social Cognitive Theory; Elaboration Likelihood Theory; Transtheoretical Model; Diffusion of Health Promotion Innovation.

## References

[1] Laverack G. (2017). The challenge of the ‘art and science’ of health promotion. Challenges.

[2] Xiaoyan O., Diego O., Alireza S., Alessandro F., Filippo M. (2017). Limited individual attention and online virality of low-quality information. Nat. Hum. Behav..

[3] Falkinger J. (2007). Attention economies. J. Econ. Theory.

[4] Thomson K., Hillier-Brown F., Todd A., Mcnamara C., Huijts T., Bambra C. (2018). The effects of public health policies on health inequalities in high-income countries: An umbrella review. BMC Public Health.

[5] Baum F., Fisher M. (2014). Why behavioral health promotion endures despite its failure to reduce health inequities. Sociol. Health Illn..

[6] Frieden T.R. (2014). Six components necessary for effective public health program implementation. Am. J. Public Health.

[7] Gill T., Boylan S. (2012). Public health messages: Why are they ineffective and what can be done?. Curr. Obes. Rep..

[8] Jepson R.G., Harris F.M., Platt S., Tannahill C. (2010). The effectiveness of interventions to change six health behaviours: A review of reviews. BMC Public Health.

[9] Debarr K. (2004). A Review of Current Health Education Theories. Calif. J. Health Promot..

[10] Rosenstock I.M., Strecher V.J., Becker M.H. (1998). Social Learning Theory and the Health Belief Model. Health Educ. Q..

[11] Ajzen I. (1991). The theory of planned behavior. Organ. Behav. Hum. Decis. Process..

[12] Bandura A. (2001). Social cognitive theory: An agentive perspective. Annu. Rev. Psychol..

[13] Petty R.E., Caciopo J.T. (1986). The elaboration likelihood model of persuasion. Adv. Exp. Soc. Psychol..

[14] Caplan G. (1974). Support. Systems and Community Mental Health.

[15] Prochaska J.O., Diclemente C.C. (1983). Stages and processes of self-change of smoking: Toward an integrative model of change. J. Consult. Clin. Psychol..

[16] Rogers E.M. (1995). Diffusion of Innovations.

[17] Singhal A. (2002). A Theoretical agenda for entertainment-education. Commun. Theory.

[18] McLeroy K., Bibeau D., Steckler A., Glanz K. (1988). An ecology perspective on health promotion programs. Health Educ. Q..

[19] Langille J., Rodgers W.M. (2010). Exploring the influence of a Social Ecological Model on school-based physical activity. Health Educ. Behav..

[20] Stokols D., Allen J., Bellingham R.L. (1996). The social ecology of health promotion: Implications for research and practice. Am. J. Health Promot..

[21] Williams O., Noble J.M. (2008). Hip-Hop Stroke: A stroke educational program for elementary school children living in a high-risk community. Stroke.

[22] Williams O., DeSorbo A., Noble J., Gerin W. (2012). Child Mediated Stroke Communication: Findings from Hip Hop Stroke. Stroke.

[23] Williams O., Leighton-Herrmann E., DeSorbo A., Hecht M., Hedmann M., Huq S., Gerin W., Chinchilli V., Ogedegbe G., Noble J. (2015). Hip Hop Stroke: Study protocol for a randomized controlled trial to address stroke literacy. J. Clin. Trials.

[24] Williams O., Leighton-Hermann E., Hecht M., DeSorbo A., Gerin W., Hedmann M., Shelton R., Tolchin B., Noble J. (2016). Child Mediated Health Communication: A conceptual framework for increasing stroke literacy in hard to reach populations. J. Health Disparities Res. Pract..

[25] Williams O., Leighton-Herrmann E., Teresi J., Eimicke J.K., Kong J., Ogedegbe G., Noble J. (2018). Improving community stroke preparedness in the HHS (Hip-Hop Stroke) randomized clinical trial. Stroke.

[26] Hayslip B., Kaminski P.L. (2005). Grandparents raising their grandchildren: A review of the literature and suggestions for practice. Gerontologist.

[27] Spence J.C., Lee R.E. (2003). Toward a comprehensive model of physical activity. Psychol. Sport Exerc..

[28] Lewis M.A., Fitzgerald T.M., Zulkiewicz B., Peinado S., Williams P. (2017). Identifying synergies in multilevel interventions. Converg. Strategy. Health Educ. Behav..

[29] Golden S.D., Earp J.A.L. (2012). Social ecological approaches to individuals and their contexts: Twenty years of health education & behavior health promotion interventions. Health Educ. Behav..

[30] Batra R., Keller P.A., Strecher V.J. (2012). Leveraging Consumer Psychology for Effective Health Communications: The Obesity Challenge.

[31] Stuckey H.L., Nobel J. (2010). The connection between art, healing, and public health: A review of current literature. Am. J. Public Health.

[32] Yom-Tov E., Shembekar J., Barclay S., Muenning P. (2018). The effectiveness of public health advertisements to promote health: A randomized-controlled trial on 794,000 participants. Digit. Med..

[33] Kreuter M., Lukwago S., Bucholtz R.D., Clark E.M., Sanders-Thompson V. (2003). Achieving cultural appropriateness in health promotion programs. Health Educ. Behav..

[34] Resnicow K., Baranowski T., Ahluwahlia J.S., Braithwaite R.L. (1999). Cultural sensitivity in public health: Defined and demystified. Ethn. Dis..

[35] Mann C. (2011). Behaviour Changing Campaigns: Success and Failure Factors. Transparency International. https://www.transparency.org/whatwedo/answer/behaviour_changing_campaigns_success_and_failure_factors.

[36] Jacobs J.A., Clayton P.F., Dove C., Funchess T., Jones E., Perveen G., Skidmore B., Sutton V., Worthington S., Baker E.A. (2012). A survey tool for measuring evidence-based decision- making capacity in public health agencies. BMC Health Serv. Res..

[37] Baldwin S., Ching Y.H. (2016). Interactive storytelling: Opportunities for online course design. Technol. Trends.

[38] Lehmann S., Murray M.M. (2005). The role of multisensory memories in unisensory object discrimination. Cogn. Brain Res..

[39] Murray M.M., Michel C.M., Grave de Peralta R., Ortigue S., Brunet D., Gonzalez Andino S., Schnider A. (2004). Rapid discrimination of visual and multisensory memories revealed by electrical neuroimaging. Neuroimage.

[40] Shams L., Seitz A. (2008). Benefits of multisensory learning. Trends Cogn. Sci..

[41] Xie Y., Bian C., Li M. (2017). Semantic congruent audiovisual integration during the encoding stage of working memory: An ERP and sLORETA study. Sci. Rep..

[42] Elder R.S., Krishna E.A. (2010). The effects of advertising copy on sensory thoughts and perceived taste. J. Consum. Res..

[43] Shi Z., Müller H.J. (2013). Multisensory perception and action: Development, decision-making, and neural mechanisms. Front. Integr. Neurosci..

[44] Quak L., London R.E., Talsma D. (2015). A multisensory perspective of working memory. Front. Hum. Neurosci..

[45] Mayer K.M., Yildiz I.B., Macedonia M., von Kriegstein K. (2015). Visual and motor cortices differentially support the translation of foreign language words. Curr. Biol..

[46] Shimojo S., Shams L. (2001). Sensory modalities are not separate modalities: Plasticity and interactions. Curr. Opin. Neurobiol..

[47] Voto D., Viñas L.M., D’Auria L. Multisensory interactive installation. Proceedings of the Sound and Music Computing ’05, XV CIM.

[48] Seitz A., Kim R., Shams L. (2005). Sound Facilitates Visual Learning. Curr. Biol..

[49] Steslow D.M., Gardner C. (2011). More than one way to tell a story: Integrating storytelling into your law course. J. Leg. Stud. Educ..

[50] Heikkilä J. (2014). Audiovisual semantic congruency during encoding enhances memory performance. Exp. Psychol..

[51] Delp C., Jones. J. (1996). Communicating information to patients: The use of cartoon illustrations to improve comprehension of instructions. Acad. Emerg. Med..

[52] Houts P.S., Witmer J.T., Egeth H.E., Loscalzo M.J., Zabora J.R. (2001). Using pictographs to enhance recall of spoken medical instructions II. Patient Educ. Couns..

[53] George S., Moran E., Duran N., Jenders R.A. (2013). Using animation as an information tool to advance health research literacy among minority participants. Amia Annu. Symp. Proc..

[54] Clift S. (2012). Creative arts as a public health resource: Moving from practice-based research to evidence-based practice. Perspect. Public Health.

[55] Padilla A.M., Borsato G.N., Suzuki L.A., Ponterotto J.G. (2008). Issues in culturally appropriate psychoeducational assessment. Handbook of Multicultural Assessment: Clinical, Psychological, and Educational Applications.

[56] Lewis N.A., Oyserman D. (2016). Using identity-based motivation to improve the nation’s health without breaking the bank. Behav. Sci. Policy.

[57] Kreuter M., Mcclure S. (2004). The role of culture in health communication. Annu. Rev. Public Health.

[58] Oyserman D., Smith G.C., Elmore K. (2014). Identity-Based Motivation: Implications for health and health disparities. J. Soc. Issues.

[59] Norman C.D. (2012). Social media and health promotion. Glob. Health Promot..

[60] Jane M., Hagger M., Foster J., Ho S., Pal S. (2018). Social media for health promotion and weight management: A critical debate. BMC Public Health.

[61] Pew Research Center—Social Media Use in 2018. http://www.pewinternet.org/2018/03/01/social-media-use-in-2018/.

[62] Lewis J.J. (2017). Use of social media for the delivery of health promotion on smoking, nutrition, and physical activity: A systematic review. Lancet.

[63] Welch V., Petkovic J., Pardo J., Rader T., Tugwell P. (2016). Interactive social media interventions to promote health equity: An overview of reviews. Health Promot. Chronic Dis. Prev..

[64] Berger J., Prinstein M.J., Dodge K.A. (2008). Identity-signalling, social influence, and social contagion. Understanding Peer Influence in Children and Adolescents.

[65] Berger J., Heath C. (2005). Idea habitats: How the prevalence of environmental cues influences the success of ideas. Cogn. Sci..

[66] Berger J., Rand L. (2008). Shifting signals to help health: Using identity signaling to reduce risky health behaviors. J. Consum. Res..

[67] Rimal R.N., Real K. (2005). How behaviors are influenced by perceived norms: A test of the theory of normative social behavior. Commun. Res..

[68] Templeton E.M., Stanton M.V., Zaki J. (2016). Social norms shift preferences for healthy and unhealthy foods. PLoS ONE.

[69] Cross I. (2008). Musicality and the human capacity for cultures. Music. Sci..

[70] Murray N.M., Murray S.B. (1996). Music and lyrics in commercials: A cross-cultural comparison between commercials run in the Dominican Republic and in the United States. J. Advert..

[71] Schlosser M.J., Aoyagi N., Fulbright R.K., Gore J.C., McCarthy G. (1998). Functional MRI studies of auditory comprehension. Hum. Brain Mapp..

[72] Morrison J., Demorest S.M., Aylward E.H., Cramer S.C., Maravilla K.R. (2003). FMRI investigation of cross-cultural music comprehension. NeuroImage.

[73] North A.C., Mackenzie L.C., Law R.M., Hargreaves D.J. (2004). The effects of musical and voice “fit” on responses to advertisements1. J. Appl. Soc. Psychol..

[74] Yeoh P.S., North A.C. (2010). The Effect of musical fit on consumers’ memory. Psychol. Music.

[75] Brownson R.C., Fielding J.E., Maylahn C.M. (2013). Evidence-based decision making to improve public health practice. Front. Public Health Serv. Syst. Res..

[76] Heath C., Heath D. (2007). Made to Stick: Why Some Ideas Survive and Others Die.

[77] Berger J. (2013). Contagious: Why Things Catch On.

[78] Milkman K., Berger J. (2014). The Science of sharing and the sharing of science. Proc. Natl. Acad. Sci. USA.

[79] Brownson R.C., Chriqui J.F., Stamatakis K.A. (2009). Understanding evidence-based public health policy. Am. J. Public Health.

[80] Burns P., Rohrich R., Chung K. (2011). The levels of evidence and their role in evidence-based medicine. Plast. Reconstr. Surg..

[81] Rychetnik L., Hawe P., Waters E., Barratt A., Frommer M. (2004). A glossary for evidence based public health. J. Epidemiol. Community Health.

[82] Bernal G., Bonilla J., Bellido C. (1995). Ecological validity and cultural sensitivity for outcome research: Issues for the cultural adaptation and development of psychosocial treatments with Hispanics. J. Abnorm. Child. Psychol..

[83] Lee J.K., Hecht M.L., Miller-Day M., Elek E. (2011). Evaluating mediated perception of narrative health messages: The perception of Narrative Performance Scale. Commun. Methods Meas..

[84] Miller M., Hecht M., Stiff J. (1998). An exploratory measurement of engagement with live and film media. J. Ill. Speech Theatre Assoc..

[85] Naples M.J. (1997). Effective frequency—Then and now. J. Advert. Res..

[86] DeSorbo A., Noble J., Shafer M., Gerin W., Williams O. (2013). The use of an audience response system in an elementary school-based health education program. Health Educ. Behav. J..

[87] Simmons C., Noble J.M., Leighton-Herrmann E., Hecht M.F., Williams O. (2017). Community-level measures of stroke knowledge among children: Findings from Hip Hop Stroke. J. Stroke Cerebrovasc. Dis..

[88] Saver J.L. (2005). Time is brain—Quantified. Stroke.

